# A Military-Centered Approach to Neuroprotection for Traumatic Brain Injury

**DOI:** 10.3389/fneur.2013.00073

**Published:** 2013-06-12

**Authors:** Deborah A. Shear, Frank C. Tortella

**Affiliations:** ^1^Branch of Brain Trauma Neuroprotection and Neurorestoration, Center for Military Psychiatry and Neuroscience, Walter Reed Army Institute of Research, Silver Spring, MD, USA

**Keywords:** TBI biomarkers, combination drug therapy, isobolographic, pre-clinical, neuroprotective agents

## Abstract

Studies in animals show that many compounds and therapeutics have the potential to greatly reduce the morbidity and post-injury clinical sequela for soldiers experiencing TBI. However, to date there are no FDA approved drugs for the treatment of TBI. In fact, expert opinion suggests that combination therapies will be necessary to treat any stage of TBI recovery. Our approach to this research effort is to conduct comprehensive pre-clinical neuroprotection studies in military-relevant animal models of TBI using the most promising neuroprotective agents. In addition, emerging efforts incorporating novel treatment strategies such as stem cell based therapies and alternative therapeutic approaches will be discussed. The development of a non-surgical, non-invasive brain injury therapeutic clearly addresses a major, unresolved medical problem for the Combat Casualty Care Research Program. Since drug discovery is too expensive to be pursued by DOD in the TBI arena, this effort capitalizes on partnerships with the Private Sector (Pharmaceutical Companies) and academic collaborations (Operation Brain Trauma Therapy Consortium) to study therapies already under advanced development. Candidate therapies selected for research include drugs that are aimed at reducing the acute and delayed effects of the traumatic incident, stem cell therapies aimed at brain repair, and selective brain cooling to stabilize cerebral metabolism. Each of these efforts can also focus on combination therapies targeting multiple mechanisms of neuronal injury.

## Background

Historically, combat-related traumatic brain injury (TBI) has been one of the leading causes of military casualties, responsible for 20–25% of battle-incurred injuries in previous conflicts and accounting for upwards of 42% of combat-related deaths that occur “after” reaching a surgical ward (Arnold and Cutting, 1978; Leedham et al., 1993; Salazar et al., 1995; Jevtic et al., 1996; Owens et al., 2008). More recent epidemiological data generated from the current conflicts in Iraq and Afghanistan indicates that up to 30% of combat-related trauma occurs in the head and neck region and that the vast majority (over 80%) of these casualties result from blast explosion (Owens et al., 2008). Explosive devices (i.e., improvised explosive devices (IEDs), propelled grenades, mortars, mines, bombs etc) accounted for 76% of U.S. fatalities in Iraq from June 2006–December 2006 alone, demonstrating a 20% increase over blast fatalities in 2004. In large part, this is due to the fact that enemy use of IEDs has become increasingly more deadly with larger fire balls and more explosive power causing increased fragmentation (Schreiber et al., 2008).

While advances in body armor, helmets, and clinical advanced trauma life support measures have lead to a significant decrease in mortality on the battlefield (Young and Andrews, 2008) an increasing number of these patients are facing a lifetime of cognitive and physical disabilities. In 2003, over 40% of TBI survivors had a TBI related disability one year after injury (Corrigan et al., 2010). Not only do people with TBI face disability, TBIs have also been shown to increase long-term mortality and reduce life expectancy. Further, TBI is associated with the increased incidences of seizures, sleep disorders, neurodegenerative diseases (e.g., Alzheimer’s disease, Parkinson’s disease, and epilepsy), neuroendocrine dysregulation, and psychiatric diseases (Masel and Dewitt, 2010; Smith et al., 2013).

Further analysis into the mechanisms of combat-related moderate/severe TBI indicates that *over 70% of blast-induced moderate to severe TBI are confounded by a penetrating injury to the brain* (Bell et al., 2009). These data come from a 5-year retrospective study (2003–2008) conducted at the National Naval Medical Center and Walter Reed Army Medical Center which reported that over half (229/408) of neurosurgical casualties evacuated from Theater had sustained a TBI from blast events and that 71% of these blast TBI victims also suffered penetrating TBIs (PTBI). From the total population, 40% (163/408) presented with a blast/PTBI whereas only 16% presented with a blast/closed-head TBI (66/408). Gunshot inflicted-PTBI accounts for an additional 13% of this patient segment. Overall, these data indicate that combat blast encounters resulting in moderate-severe TBI are more likely to have a penetrating rather than closed-head injury (Masel et al., 2012).

Although severe TBI represents the most significant life-threatening trauma, the vast majority of non-fatal TBIs (>80%) have been classified as “mild” (mTBI) typically caused by closed-head concussion^1^. It has been estimated that up to 28% of U.S. military personnel sustained at least one concussive mTBI event while deployed in Iraq and Afghanistan (Warden, 2006). In fact, the extremely high incidence of which concussive mTBI has occurred in our soldiers has defined this combat wound as the “signature injury” of these wars (Elder and Cristian, 2009). Further, combat troops may experience increased risk of exposure to more than one concussion or mTBI in a short timeframe, the cumulative effects of which can produce long-lasting neuropsychological disorders including physical, mental, emotional, and cognitive impairments and may place our returning soldiers at increased risk for PTSD and/or neurodegenerative disorders including chronic traumatic encephalothapy (CTE) (MacGregor et al., 2011; Goldstein et al., 2012; McKee et al., 2013). Critically, TBI in military personnel is not limited to combat situations (MacGregor et al., 2012). The most recent epidemiological data from the Defense and Veterans Brain Injury Center (see text footnote 1) and the Armed Forces Health Surveillance Center (AFHSC, 2013) estimates that over 80% of military-related TBI occurs in non-deployed environments. Therefore, even in times of peace, TBI will remain a significant medical concern for the military and poses an even greater economic concern for the military as service members retire and face potential long-term consequences from brain injury.

Listed in the Guideline for Management of Severe TBI (Brain Trauma Foundation et al., 2007) are at least 14 emergency room (ER) approaches for managing severe TBI in the neuro-intensive care unit. These include, but are not limited to, hyperventilation, monitoring intracranial pressure, anti-seizure prophylaxis, infection prophylaxis, and sedation. The primary goal of these ER managements is to achieve stabilization of all vital systems and allow further assessment and treatment, particularly neuroprotective therapies that can improve neurological, motor, and cognitive functions. Presently, no drug therapy is approved as standard of care for the treatment of TBI.

Our primary mission under the directive of the Combat Casualty Care Research Program (CCCRP) is to conduct pre-clinical studies of neuroprotection therapies aimed at mitigating TBI. During the past decade and under the directive of the CCCRP, our research team established a rodent model of penetrating ballistic-like brain injury (PBBI) which was designed to model the permanent injury tract created by the path of a ballistic and the large temporary cavity generated by the ballistic energy dissipated from the penetrating object (Williams et al., 2005, 2006a,b). The PBBI model can be adjusted to represent any penetrating projectile that carries either a low (9 mm and/or fragments) or high (7.62 round = AK-47, M-16, etc.) velocity capable of producing a leading pressure or shock wave to the brain.

The unilateral frontal PBBI model has been extensively characterized and captures the acute neuropathological events associated with penetrating TBI, including lacerated brain damage, intracerebral hemorrhage, increased intracranial pressure, axonal degeneration, up-regulation of pro-inflammatory cytokines, and electrocortical disturbances (Williams et al., 2005, 2006a,b). It also produces reliable and enduring motor and cognitive deficits (Shear et al., 2010, 2011; Mountney et al., 2013) and electrophysiological insults (Lu et al., 2011, 2013), and has proven useful for assessing neuroprotective effects of promising therapeutic interventions (Lu et al., 2009b; Shear et al., 2009; Wei et al., 2009; Deng-Bryant et al., 2012). Specifically, to date we have reported evidence indicating that DM, a potent NMDA antagonist and sigma-1 receptor ligand, and NNZ-2566, a glypromate analog, and novel neuroprotective compound (Neuren Pharmaceutical Inc.) are effective in promoting functional recovery following PBBI (Lu et al., 2009a; Shear et al., 2009). We have also demonstrated that NNZ-2566 protects against PBBI-induced up-regulation of pro-inflammatory cytokines (Wei et al., 2009). Our pre-clinical NNZ-2566 data from the PBBI model has directly contributed to the recent clinical advancement of this compound into a multi-center Phase II trial for moderate-severe TBI.

More recently our research team took on the task of developing a rodent model of closed-head concussive mTBI. Our approach to this model was to produce molecular changes in the brain and alterations in behavior that would be indicative of an mTBI without making any surgical incisions and without producing any gross morphological damage like skull fracture or intracerebral hemorrhage. We recently reported the proof-of-concept development of a projectile concussive impact (PCI) injury model that produces a true closed-head concussive event resulting in significant cellular, molecular, and sensorimotor changes with no evidence of gross contusional injury (Chen et al., 2012). Studies currently underway include longitudinal and multi-modal designs to fully characterize the neuromotor, cognitive, emotional, and neuropathological evidence of concussive brain injury using the PCI model. The overall goal is to develop a more thorough understanding of the changes taking place at a cellular level following a single or multiple concussive events, for the purpose of evaluating putative therapeutic interventions.

## Drug Discovery and Development

Our approach to drug discovery and development consists of our Cooperative Research and Development Agreement (CRADA) partnerships with major pharmaceutical companies and our ongoing collaborative effort with the Operation Brain Trauma Therapy (OBTT) Consortium (Kochanek et al., 2011). Novel drug discovery and development in partnership with private pharmaceutical companies represents a critical component of our TBI/Neuroprotection Research Program. Our CRADA partnerships give us access to lead neuroprotective drug candidates keeping us at the drug discovery forefront. Importantly, our Program has a long history of successful collaborations with drug companies and our efforts have directly resulted in two clinical trials: Phase I clinical trial on MLN 519 for stroke (terminated after successful Phase I), and the Phase II clinical trial on NNZ-2566 for moderate and severe TBI (*INTREPID-2566*, ongoing). We currently have CRADAs with several private pharmaceutical companies to conduct studies assessing novel compounds in our PBBI model that target a number of different TBI mechanisms. The basic premise of this work is to first establish proof-of-principle therapeutic efficacy for a novel CRADA-sponsored drug in the PBBI model and evaluate the full dose-response monotherapy profile of the most promising drugs for potential consideration as a candidate for advanced combination drug therapy studies. For the combination therapy studies, we focus primarily on the most promising neuroprotective drugs described in the TBI literature that either have already been approved by the FDA for other clinical indications, or are in the process of being advanced into clinical trials.

The OBTT is a multi-center consortium developed with the primary purpose of rapidly screening potential TBI therapies and TBI biomarkers and translating them ultimately to combat casualty care (Kochanek et al., 2011). The inception of the OBTT Consortium was predicated on the observation that the mechanistic-based approach to TBI research, which dominated the field over the past two decades, has hindered the rapid advancement of new therapies to the clinic. The primary purpose of the OBTT Consortium was to address this issue by screening drugs of high interest across three TBI rodent models with the idea the best drug(s) would be subjected to advanced testing in a TBI pig model with the ultimate goal to facilitate the rapid translation of the most promising therapies to the clinic (Kochanek et al., 2011).

## Alternative Therapeutic Approaches for TBI

### Neural stem cell transplantation

We have previously demonstrated that human amnion-derived progenitor (AMP) cell transplantation protects against injury-induced neuropathology and motor deficits in the PBBI model (Chen et al., 2009, 2011). However, the functional recovery observed in those studies occurred too rapidly (within 1 week post-injury) to be attributed to any host-graft functional connectivity. This suggested the transplanted cells may be mediating functional recovery through a variety of mechanisms associated with inducing neuroplasticity, including the sustained secretion of cytokines/growth factors which are abundant in amnion-derived cellular cytokine solution (ACCS).

Amnion-derived cellular cytokine solution contains physiological concentrations of dozens of factors, many of which are involved in the wound healing cascade, including the growth factors TGF-B2 and PDGF and the metalloproteinase inhibitors Timp-1, Timp-2 (Steed et al., 2008). Accordingly, ACCS has been shown to have a significant effect in a variety of burn and incisional wound healing models (Franz et al., 2008; Uberti et al., 2009; Payne et al., 2010). Our most recent work has demonstrated that chronic intracerebroventricular infusion of ACCS promoted significant protection against PBBI-induced neuropathology and motor abnormalities (Deng-Bryant et al., 2012). However, in that study ACCS was not effective in reducing cognitive deficits, nor was it effective when delivered intravenously, indicating that blood brain barrier (BBB) permeability may be a mitigating factor.

### Selective brain cooling

Research focused on elucidating the effects of mild-to-moderate therapeutic hypothermia on severe TBI has consistently demonstrated therapeutic benefits in pre-clinical studies. However, the majority of these studies have utilized whole-body cooling techniques, which may pose an increased clinical risk of adverse side effects including coagulopathy, hypotension, and infectious pneumonia in TBI patients (Shiozaki et al., 2001; Bernard et al., 2002; Milhaud et al., 2005; Hemmen and Lyden, 2007; Sydenham et al., 2009). Clinically, these adverse effects have raised serious concerns for the application of therapeutic hypothermia, particularly when treating patients with severe hemorrhage (Romlin et al., 2007). In order to maximize the potential benefits of hypothermia while minimizing the potential for adverse effects, we developed a novel method of selective brain cooling (SBC) using bilateral common carotid artery (CCA) cooling cuffs that can achieve rapid and sustained reductions in core brain temperature while maintaining normal (37 °C) body temperature (Wei et al., 2008). We recently published results demonstrating the therapeutic efficacy of SBC in the PBBI model including significant reductions in acute post-injury measures of intracranial pressure, brain edema, BBB permeability, and lesion volume (Wei et al., 2011).

## Combination Drug Therapy Development for TBI

Research in the TBI field has generated a plethora of data demonstrating significant pre-clinical therapeutic efficacy from over 130 drugs, which in turn has resulted in over 20 Phase II/III clinical trials over the past two decades. However, this approach has yet to succeed in producing a single therapy which has demonstrated clinically significant neuroprotective efficacy in TBI (Margulies et al., 2009). One major reason cited for these disappointing outcomes is that monotherapy approaches, that target single or limited mechanisms, are simply not adequate to address the complex and dynamic milieu of the injured brain. In recognition of the limitations of the monotherapy approach to treating TBI, increased attention is now being directed toward developing combination therapeutic strategies. This issue was addressed by a panel of TBI experts and called for a revisiting of the most promising neuroprotective agents and challenged the TBI research community to develop step-by-step strategies for pre-clinical and clinical research on combination drug therapy development (Margulies et al., 2009).

A more recent focus of our military-focused research program was to address the challenge of combination drug therapy development. Our approach to this problem was to apply the *isobolographic method* of combination drug therapy development to our TBI neuroprotection studies. The isobolographic method represents the industry “gold standard” pharmacological approach for detecting drug–drug interactions (Tallarida, 2012). This step-by-step statistical method was originally introduced in 1953 (Loewe, 1953) and has since been developed and extended by Tallarida (2012) and others, and applied to numerous pre-clinical and clinical analyses of combination data. For example, Dr. Tallarida has published>80 peer-reviewed papers and several textbooks on this subject matter and his isobolographic analysis guided the pre-clinical and clinical studies that led to a patent (U.S. 5,336,691) for the analgesic combination of tramadol and acetaminophen (Tallarida and Raffa, 1996) and to the subsequent development of the product Ultracet ©that is a synergistic combination of the two drugs.

Overall, the key criteria for a successful pre-clinical combination therapy is to (1) improve the therapeutic effects achieved via monotherapy through the *synergistic* interaction of two or more drugs administered in combination and (2) to effectively lower the risk of adverse effects by using sub-threshold doses of the individual drugs in combination (Tallarida, 2012). Thus, the strength in the isobolic approach to combination therapy development lies in its ability to distinguish between additive and synergistic effect of drug-pairs. Of equal importance is that the isobolic approach provides a well-established statistical framework for identifying sub-additive or potentially antagonistic effects of drug-pairs that could be indicative of contraindication.

## Prognostic and Theragnostic Value of TBI Biomarkers

In addition to treatment, of paramount concern to the military is the lack of a rapid, objective test, or criteria for clinical diagnosis of mTBI/concussion and/or a means of tracking the chronic evolution of the TBI across all levels of injury severity. Mild cases of TBI are often under-diagnosed and under-reported, and often escape detection by brain imaging. In contrast, moderate and severe cases of TBI may be easier to detect, accurate prognostic indications and long-term therapeutic management remains a challenge.

Overall, numerous efforts across the TBI field are attempting to solve this problem and much of these efforts are reviewed in this special edition of Frontiers. TBI-specific biomarkers that have been established in experimental models of TBI and implicated in human clinical TBI studies include include S100B, glial fibrillary acidic protein (GFAP), Ubiquitin C-terminal hydrolase L1 (UCH-L1), Neuron Specific Enolase (NSE), Alpha-II spectrin breakdown products (SBDP), and Tau (Brophy et al., 2011; Mondello et al., 2011, 2012a,b). Of these, S100B has been shown to upregulate in response to other trauma in the absence of TBI and thus its diagnostic value to the military may be limited (Bloomfield et al., 2007). In contrast, serum GFAP levels have been reported to show both good specificity and sensitivity to TBI (Mondello et al., 2011, 2012a; Papa et al., 2012a) and serum levels of GFAP breakdown products have been correlated with brain imaging studies of mild and moderate TBI suggesting that GFAP may serve as a marker for mTBI (Brophy et al., 2011). Research has also shown UCH-L1 (a marker of neuronal damage) is significantly increased in the CSF of TBI patients during the acute post-injury phase and has been correlated with negative outcome (Brophy et al., 2011; Papa et al., 2012b). Alpha-II spectrin is located primarily in axons and presynaptic terminals of neurons (Riederer et al., 1986) and is cleaved by calpain and caspase 3 (Nath et al., 1996; Wang et al., 1998) representing both necrotic and apoptotic mechanisms. SBDPs have been detected in animals in both brain and CSF after moderate CCI injury (Ringger et al., 2004) and brain tissue following mild FPI (McGinn et al., 2009).

However, there still remains a critical need for research on TBI-specific biomarkers that are sensitive to the chronic evolution of TBI neuropathology and that can reliably measure the therapeutic efficacy of a particular drug. Collectively, as regards our gaps in treatment and diagnosis, there is an increased demand for pre-clinical TBI research addressing these concerns, particularly across animal models of mild, moderate and severe TBI.

## Conflict of Interest Statement

The authors declare that the research was conducted in the absence of any commercial or financial relationships that could be construed as a potential conflict of interest.

## Footnotes

^1^http://www.dvbic.org/dod-worldwide-numbers-tbi
